# Effects of ophidiomycosis on movement, survival, and reproduction of eastern foxsnakes (*Pantherophis vulpinus*)

**DOI:** 10.1038/s41598-024-54568-x

**Published:** 2024-02-28

**Authors:** Rachel M. Dillon, James E. Paterson, Pilar Manorome, Kyle Ritchie, Leonard Shirose, Emily Slavik, Christina M. Davy

**Affiliations:** 1 Environmental and Life Sciences Program, Trent University, Peterborough, ON K9H 7B8 Canada; 2 Wildlife Research and Monitoring Section, Ontario Ministry of Natural Resources, 2Nd Flr DNA Building, 2140 East Bank Dr., Peterborough, ON K9L 1Z8 Canada; 3 Wildlife Preservation Canada, 5420 Highway 6 North, Guelph, ON N1H 6J2 Canada; 4 Department of Biology, Carleton University, 1125 Colonel By Drive, Ottawa, ON K1S 5B6 Canada; 5 Institute for Wetland and Waterfowl Research, Ducks Unlimited Canada, Stonewall, MB Canada; 6 Ontario Parks, Ontario Ministry of Environment, Conservation, and Parks, 300 Water Street, 3Rd Floor S, Peterborough, ON K9J 8M5 Canada; 7 Department of Pathobiology, University of Guelph, Guelph, ON N1G 2W1 Canada; 8 Canadian Wildlife Health Cooperative – Ontario/Nunavut, Guelph, ON N1G 2W1 Canada; 9 Lake Erie Management Unit, Ontario Ministry of Natural Resources, 320 Milo Road, Wheatley, ON N0P 2P0 Canada

**Keywords:** Eastern foxsnake, *Pantherophis vulpinus*, Ophidiomycosis, Snake fungal disease, Fitness, Body condition, qPCR, Survivorship, Behavioural ecology, Ecological epidemiology, Population dynamics

## Abstract

Ophidiomycosis (snake fungal disease) is caused by the fungal pathogen *Ophidiomyces ophidiicola*, which causes dermal lesions, occasional systemic infections, and in some cases, mortality. To better understand potential conservation implications of ophidiomycosis (i.e., population-level effects), we investigated its impacts on individual fitness in a population of endangered eastern foxsnakes (*Pantherophis vulpinus*). We tracked 38 foxsnakes over 6 years and quantified body condition, movement patterns, oviposition rates, and survival. Body condition, distance travelled, and oviposition rates were similar between snakes with and without ophidiomycosis. Interestingly, snakes that tested positive for the pathogen travelled farther, suggesting that movement through a greater diversity of habitats increases risk of exposure. Ophidiomycosis did not negatively affect survival, and most apparently infected snakes persisted in a manner comparable to snakes without ophidiomycosis. Only one mortality was directly attributed to ophidiomycosis, although infected snakes were overrepresented in a sample of snakes killed by predators. Overall, our results suggest that ophidiomycosis may have sublethal effects on eastern foxsnakes, but do not suggest direct effects on survival, ovipositioning, or viability of the study population.

## Introduction

Habitat loss, climate change, overexploitation, and the introduction of invasive species are driving rapid declines in biodiversity, especially in reptiles and amphibians^1–5^. These pressures can interact with infectious diseases to further threaten declining populations^6–8^. The number of emerging diseases caused by fungi has risen during the last two decades^7^. These include chytrid fungus (*Batrachochytrium dendrobatidis* and *B. salamandrivorans),* which have caused declines and extinctions in amphibians^9^, and *Pseudogymnoascus destructans,* the fungus that causes bat white-nose syndrome (WNS)^10^. Both these examples illustrate how fungal pathogens can have substantial, rapid impacts on biodiversity.

When an infectious disease negatively affects individual fitness and a large proportion of individuals are affected, the disease may cause population declines and strong selection for disease tolerance or resistance. For example, some populations of bats affected by WNS declined by > 95% following initial exposure to the pathogen, with strong selection for larger individuals with particular immunogenetic profiles^11–13^. Alongside direct measures of fitness (survival and reproduction)^14^, some diseases exert sublethal effects on body condition and behaviour. Lower body condition was associated with fungal colonization in house sparrows (*Passer domesticus)*^15^, and pool frogs (*Pelophylax lessonae*) infected with *B. dendrobatidis* moved shorter distances than uninfected individuals^16^.

Ophidiomycosis (snake fungal disease) is caused by the fungus *Ophidiomyces* (formerly *Chrysosporium*) *ophidiicola*^17^, and has been reported in a variety of wild snake species from the USA, Canada, and Europe^18,19^. There is some debate as to whether ophidiomycosis is an “emerging” disease because specimens collected as early as 1986 retrospectively tested positive for the fungal pathogen^20,21^. Clinical signs consistent with ophidiomycosis may be synonymous with previously described “hibernation blisters"^22,23^. Genomic analyses suggest that the fungus was introduced to North America multiple times over the last few centuries, but it is unclear whether its current distribution is stable or expanding^24^.

*Ophidiomyces ophidiicola* is found on a variety of substrates in a range of environments, and apparently infects snakes opportunistically^25^. Characteristic lesions include crusty scales, superficial pustules, and subcutaneous nodules^23,26–29^. The gold standard for diagnosis includes histopathologic confirmation of fungal hyphae or arthroconidia in lesions alongside qPCR identification of the pathogen^30^. However, this is not always feasible in field studies, which sometimes infer ophidiomycosis from observation of clinical signs^31^; or the combination of clinical signs and qPCR detection of the pathogen^32–34^. Prevalence of clinical signs and detectable pathogen loads are both higher as snakes emerge from overwintering, and lesions tend to resolve over the active season^34,35^.

Ophidiomycosis is occasionally associated with morbidity and mortality^17,26,27,36^, but mortality associated with ophidiomycosis may arise from secondary complications such as anorexia, rather than direct fungal damage^37^. Although ophidiomycosis was initially identified as a conservation concern after its description^38^, it remains unclear whether the disease is severe enough to affect population growth^19^. Mark-recapture estimates in a population of Queensnakes (*Regina septemvittata)* found no effect of ophidiomycosis on recapture probabilities (a proxy for survival)^33^. Similarly, pregnant pygmy rattlesnakes (*Sistrurus miliarius)* exhibited similar prevalence of apparent ophidiomycosis, compared to non-reproductive females^35^. Effects of the disease on individual fitness (i.e., survival and reproduction) have not been quantified in other species.

Quantifying how wildlife diseases such as ophidiomycosis might affect population growth requires a mechanistic understanding of how diseases affect the fitness of free-ranging individuals. In this study, we quantified the impacts of ophidiomycosis on individual fitness in a wild population of endangered *Pantherophis vulpinus*^39^. We hypothesized that if ophidiomycosis negatively impacts host fitness, then snakes with ophidiomycosis should have lower survival, and a lower probability of reproduction than uninfected snakes. We hypothesized that if snakes with ophidiomycosis might also exhibit lower body condition and altered movement patterns due to the energetic costs of infection and recovery.

## Methods

### Data collection

We studied an endangered population of *P. vulpinus* at Rondeau Provincial Park in Morpeth, Ontario, Canada. We previously reported seasonal fluctuations in the prevalence of *O. ophidiicola* in foxsnakes at this site, alongside observations of clinical signs consistent with ophidiomcycosis, and the resolution of clinical signs in a set of individuals tracked using radio-telemetry^34^. We returned to this dataset to explicitly test the effects of ophidiomycosis on fitness in free-ranging foxsnakes, considering two direct measurements of fitness (survival and reproduction), and two proxies (body condition and movement behaviour).

To briefly outline our previously described mark-recapture, telemetry, and disease surveillance methods^34^: from 2013–2018, we captured *P. vulpinus* by searching in suitable habitat and under a network of cover boards established at Rondeau Park. Snakes with body mass > 50 g were implanted in the field with 12 mm Passive Integrated Transponder (PIT) tags (Biomark, Inc.) using a sterile injection syringe. We transported 38 large, mature individual snakes to a qualified veterinarian (Dr. Sean Egan, Egan Fife Animal Hospital, Chatham, ON), who implanted transmitters (SI-2, HOLOHIL, Carp, Ontario, Canada; 9–11 g each) under sterile surgical conditions and with full anaesthesia (see Dillon et al.^34^ for full details). Transmitters weighed < 3% of the body mass of eligible individuals and were replaced annually as they lasted ~ 12–18 months. We removed all remaining transmitters in the summer of 2019 and released the snakes following recovery from surgery.

Snakes were tracked daily for the first week post-surgery, and then once weekly (2013–2015) or twice weekly (2016–2018). Tracking took place from the time snakes emerged from overwintering in April–May until they returned to overwintering sites (September–October). We recorded snake locations using a handheld GPS unit (± 3 m). Where possible (i.e., where a snake could be reached), we captured each tracked snake monthly to measure snout-vent length, tail length, and mass, and carefully examined them for lesions. Snakes with clinical signs (≥ 1 lesions) consistent with ophidiomycosis were considered clinically positive^35^.

In 2017 and 2018 we also collected swabs to test for the presence of *O. ophidiicola*. Following Hileman et al.^40^, we swabbed the full surface of each snake with two separate swabs (Puritan 3″ Sterile Standard Cotton Swab w/Semi-Flexible Polystyrene Handle), which were stored together in a single sample tube containing lysis buffer. We collected two additional swabs from clinically positive snakes, targeting the lesions, and stored these together in a single sample tube containing lysis buffer.

All field and surgical procedures received ethics approval from the Wildlife Animal Care Committee of the Ontario Ministry of Natural Resources and Forestry (OMNRF; protocol no. 343) and the Animal Care Committee at Trent University (protocol no. 24900), in compliance with the ARRIVE guidelines^41^. All procedures were performed in accordance with relevant guidelines and regulations, and were authorized by the OMNRF under a Fish and Wildlife Scientific Collector’s Authorization, a permit under the Ontario Endangered Species Act, and a research authorization from Ontario Parks.

### Estimating population trends from capture mark-recapture data

The hypothesis that ophidiomycosis can reduce population viability of *P. vulpinus* predicts a decline in population size over time when the disease is present. To test this prediction, we estimated population size over the study period using data from all marked individuals (n = 179) from 2013 to 2018. We estimated the abundance of *P. vulpinus* with the POPAN formulation^42^ of the Jolly–Seber open population model^43,44^, which has four parameters. The probability of observing an individual at a capture event is estimated with parameters for apparent survival (Φ) and detection probability (*p*). Two parameters model the probability of new individuals entering the population: ${\hat{\text{N}}}$, the total number of individuals available to enter the population and *p*_*ent*_, the proportion of new individuals from ${\hat{\text{N}}}$ entering the site at each event. We considered each year as a capture event and allowed Φ and *p*_*ent*_ to vary with each year. We fit the model with RMark^45^ All further analyses detailed below apply only to radio-tracked snakes.

### Detection of *O. ophidiicola* from swab samples

DNA was extracted from swab samples at the Canadian Wildlife Health Centre (CWHC) in Guelph, Ontario, and tested for the presence of *O. ophidiicola* using a validated real-time polymerase chain reaction (qPCR) assay^25^. The precision and detection limit of the assays were evaluated based on a ten-fold standard curve dilution series of gDNA from 5 to 50,000 fg of DNA. We considered *O. ophidiicola* present on a snake if DNA amplification occurred within 40 cycles (cyclic threshold (Ct)). Samples that did not amplify within 40 cycles were considered negative for *O. ophidiicola*, although we acknowledge that this category may include samples that contained fungal DNA below the detection threshold.

### Estimating effects of ophidiomycosis on survival

We compared the survival of radio-tracked *P. vulpinus* with and without clinical signs of ophidiomycosis in 2017 and 2018 (n = 17) using multi-state-multi-fate models implemented in Rmark^45^. We only included data from 2017 to 2018 in this analysis because recaptures and health assessments were performed most regularly in those two summers. For each month of tracking (May–Sept. 2017 and 2018; 10 months total), we classified snakes into one of four states: (1) Unknown (i.e., previously uncaptured, or not captured in that month), (2) Dead, (3) Clinically Positive, or (4) No Clinical Signs. Clinically positive snakes also tested qPCR positive for the pathogen at least once monthly via skin swabs (procedure described above). The multi-state model included three parameters: the monthly probability of surviving between capture events (*S*), the monthly probability of transitioning between states (ψ), and the monthly probability of detecting an animal in a capture event (*p*). This estimate (*p*) accounted for snakes that were tracked but could not be captured in a particular month (i.e., they were high in a tree, or underground). We fixed the probability of transitioning from dead to all other states to 0. We compared three models using small-sample-corrected Akaike’s Information Criterion (AIC_c_): (1) a model with different ψ estimates for each state (ψ ~ state), (2) a model with different ψ estimates for dying versus switching from No Clinical Signs to Clinically Positive (ψ ~ simple state), and (3) a model with constant ψ (the same probability of transitioning between all states; ψ ~ 1). We compared the probability of transition from Clinically Positive to Dead to the probability of transition from Clinically Positive to No Clinical Signs, or from No Clinical Signs to Dead, to test whether the probability of mortality was higher in clinically positive snakes.

To address this question in a different way, we combined our field data with CWHC necropsy findings from *P. vulpinus* found depredated or dead on the road at our study site between 2013 and 2018. We used a general linear model with a binomial error structure to determine if disease prevalence differed among snakes found live under cover boards, snakes found depredated, and snakes found dead on the road. We used Tukey pairwise comparisons to test if the proportion of snakes with ophidiomycosis was different between each pair of groups.

### Estimating the effect of ophidiomycosis on reproduction

Robust quantification of reproductive output would require counting the number of eggs laid and monitoring hatch success. Ideally, juvenile survival would also be considered. These variables could not be quantified in our study, but we were able to record the proportion of tracked female snakes that became gravid in each year. We inferred oviposition by observing absence of eggs on subsequent recaptures. We used a Fisher’s exact test to compare the observed rates of ovipositioning between female snakes with and without clinical signs.

### Estimating the effect of ophidiomycosis on body condition

We calculated body condition for each *P. vulpinus* tracked from 2013 to 2018 using a body condition index (BCI) that is a reliable indicator of fat reserves for snakes^46–48^. First, we used log-transformed mass and snout-vent length (SVL) to estimate the parameters of a linear model. We used all captures for each individual where body mass was taken. Second, we used the residuals from the log-transformed mass and SVL linear model as the BCI for all captures of all snakes. To avoid confounding effects of sex and egg development on mass, we analyzed male and female snakes separately, and excluded females in years where they became gravid. We compared BCI within each year between snakes that were infected (positive qPCR and observed clinical signs) or recovering from recent infection, and snakes that did not show clinical signs of infection in that year. We used a generalized additive mixed model (*gamm*, Package *mgcv)*^49^ on male snakes and then female snakes, respectively. We included random effects for snake identity and year and smoothed the effect of day of year on BCI for snakes with and without clinical signs of ophidiomycosis.

### Estimating the effect of ophidiomycosis on movement

We hypothesized that if snakes with ophidiomycosis were lethargic due to the infection, or spent substantially more time basking, they might move shorter distances, predicting that clinically positive snakes might have smaller home ranges (i.e., use smaller areas throughout the active season) than snakes with no clinical signs. We quantified minimum home range size by creating yearly minimum convex polygons (100% MCPs) for each individual (n = 34) based on telemetry data from 2013 to 2018, using the *adehabitatHR* package from R^50^. Snakes with < 10 relocations per year (n = 4) were removed from statistical analyses^51^. We compared the home range size of clinically positive and negative snakes using linear mixed-effects models with a random effect of individual. We repeated the model for snakes tracked in 2017–2018, to test whether home range differed between snakes that were or were not carrying the pathogen, *O. ophidiicola*.

Further, we examined the daily distance travelled (DDT) by tracked snakes to determine if ophidiomycosis increases or reduces energy investment in movement, which is tied to foraging and mate-seeking. We could not quantify the absolute distance travelled by the snakes, but comparing DDT among individuals tracked with the same frequency provides a comparable proxy for movement. We used a linear mixed model (Package *lme4)*^52^ with the DDT for each snake tracked between 2013 and 2018 (including each relocation (n = 1605) for each individual (n = 32 snakes), as a function of clinical signs of ophidiomycosis and then *O. ophidiicola* presence, using individual as a random effect.

## Results

### Estimating population trends from capture mark-recapture data

The POPAN Jolly–Seber model estimated fluctuations in population size over the study period, with a minimum estimate of of 42 snakes (95% CI 18–66) in 2019, and a maximum estimate of 104 (95% CI 60–149) in 2015 (Table 1). The model did not estimate a meaningful increase or decrease in population size, as the confidence intervals of the estimated annual abundance overlapped between all pairs of years.

**Table 1:** The estimated abundance, 95% confidence intervals, and observed number of eastern foxsnakes (*Pantherophis vulpinus;* n = 179 individuals) from 2013 to 2019 using a POPAN Jolly–Seber mark-recapture model.

Year	Abundance	Lower confidence level	Upper confidence level	Observed
2013	91	49	133	41
2014	64	34	95	29
2015	104	60	149	48
2016	68	38	98	28
2017	83	46	121	37
2018	58	30	87	28
2019	42	18	66	19

### Estimating effects of ophidiomycosis on survival

**Figure 1:**
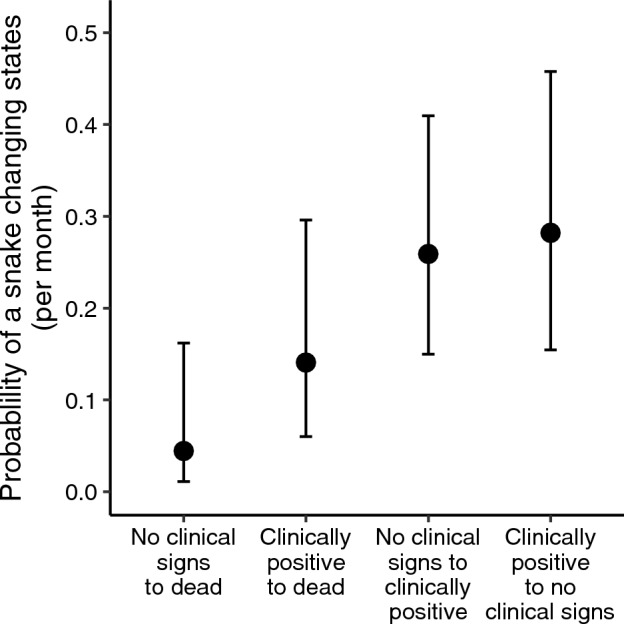
Multi-state-multi-fate model predictions of the monthly probability of snakes moving among four states. State transitions include mortality of snakes with no clinical signs of ophidiomycosis (“No clinical signs to dead”), mortality of snakes with clinical signs of ophidiomycosis (“Clinically positive to dead”), development of clinical signs in snakes that previously exhibited none (No clinical signs to clinically positive), and full resolution of gross clinical signs in snakes on which they were previously observed (Clinically positive to no clinical signs). Error bars represent 95% confidence intervals.

The most-supported multi-state model comparing survival and state transitions for snakes estimated different probabilities of transitioning between alive and dead than transitioning between clinically positive and no clinical signs (Table 2). The simple state model was more supported than the model with different survival probabilities for snakes with and without lesions. The model with different transition probabilities for all state transitions predicted that *P. vulpinus* with lesions were not more likely to die (14.1% per month, 95% CI 6.0–29.6%) than snakes without lesions (4.4% per month, 95% CI 1.1–16.2%; Fig. 1). Snakes had similar predicted probabilities of transitioning from no clinical signs to clinically positive (25.9% per month, 95% CI 15.0–41.0%), as transitioning from clinically positive to no clinical signs (28.2% per month, 95% CI 15.4–45.7%).

**Table 2:** Multi-state models for free-ranging eastern foxsnake (*Pantherophis vulpinus;* n = 17) exposed to *Ophidiomyces ophidiicola*, ranked by model weight.

Model	k	AIC_c_	ΔAIC_c_	*w*	Deviance
*S*(~ 1)*p*(~ 1)ψ(~ Simple State)	3	206.29	0.00	0.74	157.08
*S*(~ 1)*p*(~ 1)ψ(~ State)	5	208.45	2.17	0.25	154.94
*S*(~ 1)*p*(~ 1)ψ(~ 1)	2	216.12	9.83	0.01	169.02

Simple State = transitions to Dead versus transitions between No Clinical Signs and Clinically Positive”; State = all unique transitions among states. Variables: *S* = survival fixed to 1, *p* = probability of detecting the snake, ψ = probability of transitioning among No Clinical Signs, Clinically Positive, and Dead states (transitions from Dead state fixed to 0). k = number of parameters, AIC_c_ = small-sample-corrected Akaike’s information criterion, *w* = model weight.

**Figure 2:**
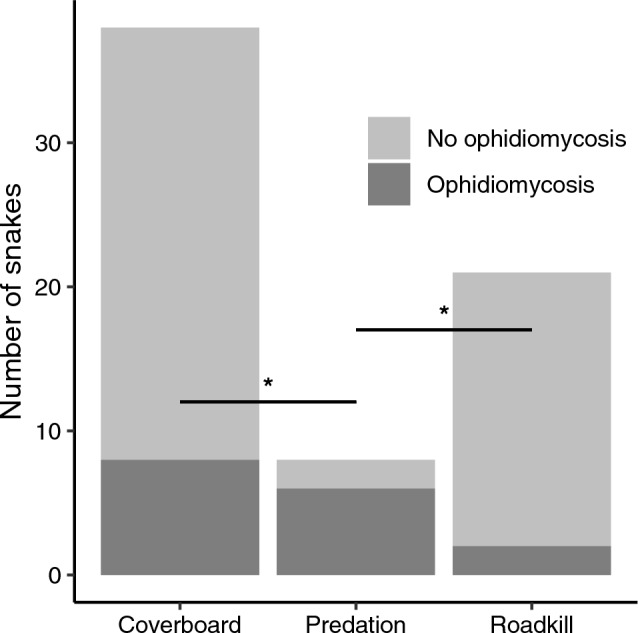
Prevalence of ophidiomycosis in live (coverboard detections; n = 38), depredated (n = 8), and road-killed (n = 21) *Pantherophis vulpinus* at Rondeau Provincial Park, Ontario (2017–2018). * indicates pairwise differences in the proportion of snakes with ophidiomycosis *p* < 0.05.

The proximate causes of death we observed were road mortality and depredation. Live *P. vulpinus* found under cover boards had similar prevalence of ophidiomycosis (8/38, 21%) compared to *P. vulpinus* found dead on the road (2/21, 10%; z = 1.10, *p* = 0.51). Depredated *P. vulpinus* had higher ophidiomycosis prevalence (6/8, 75%) than live *P. vulpinus* sampled under coverboards (z = 2.67, *p* = 0.02), or than snakes found dead on the road (z = 3.03, *p* = 0.01; Fig. 2).

### Effects of ophidiomycosis on reproduction

We observed successful oviposition in 3 of 9 clinically positive female snakes, and in 2 of 6 female snakes that did not show clinical signs (Fisher test simulated *p* = 1.0).

### Estimating the effect of ophidiomycosis on body condition

**Figure 3:**
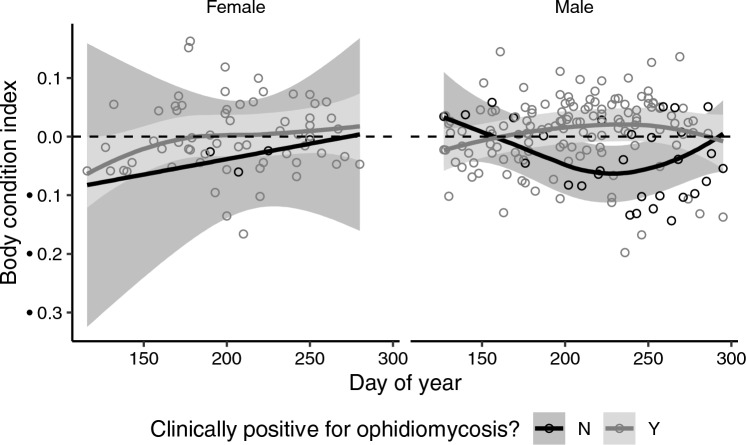
Body condition index (BCI) of eastern foxsnake (*Pantherophis vulpinus*) during the active season (May–October) was not predicted by the presence of clinical signs consistent with ophidiomycosis. Left panels shows 67 BCI estimates for 7 individual, female, non-gravid foxsnakes and the right panel shows 166 BCI estimates for 17 individual, male snakes, assigned to group (clinical signs: Y/N) based on the presence of clinical signs consistent with ophidiomycosis at the time of weighing. Solid lines are predictions from a generalized additive mixed effect model and grey ribbons represent 95% CI’s for each group.

The BCI of female *P. vulpinus* that did not become gravid increased throughout the active season, and female snakes with ophidiomycosis had similar BCI (− 0.04, 95% CI − 0.09 to 0.003) as female snakes without ophidiomycosis (BCI = − 0.08, 95% CI − 0.29 to 0.14) after emergence from overwintering (day 130; Fig. 3).

Male *P. vulpinus* with ophidiomycosis had similar body condition (BCI = − 0.02, 95% CI − 0.05 to 0.01) directly after emergence from overwintering (day 130), as males without ophidiomycosis (BCI = 0.03, 95% CI − 0.05 to 0.10; Fig. 3). In the middle of the active season (day 235), male snakes with ophidiomycosis had slightly higher BCI (0.02, 95% CI − 0.01 to 0.05) than male snakes without ophidiomycosis (− 0.06, 95% CI − 0.11 to − 0.01).

### Estimating the effect ophidiomycosis on movement

**Figure 4:**
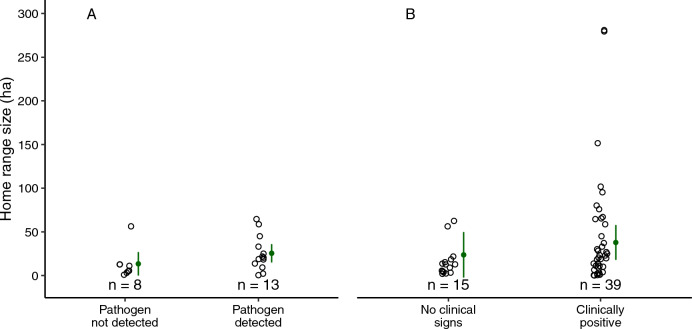
Annual home ranges of tracked eastern foxsnakes (*Pantherophis vulpinus*) (**A**) tested for *Ophidiomyces ophidiicola* (n = 21), and (**B**) assessed for clinical signs consistent with ophidiomycosis (n = 58). Each hollow point is an annual home range, and filled points and vertical bars represent model predictions and 95% confidence intervals, respectively.

**Figure 5:**
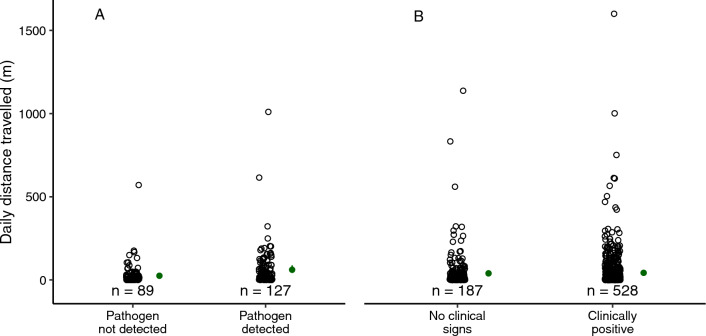
Daily distance travelled by eastern foxsnakes (*Pantherophis vulpinus*) (**A**) tested for *Ophidiomyces ophidiicola* (n = 89 steps of 8 snakes with no pathogen detected, n = 127 steps of 14 snakes with the pathogen detected), and (**B**) assessed for clinical signs consistent with ophidiomycosis (n = 187 steps of 12 snakes with no clinical signs, n = 528 steps of 30 snakes clinically positive). Each hollow point represents one movement step, and green points and vertical bars represent model predictions and 95% confidence intervals, respectively.

For *P. vulpinus* tracked from 2013 to 2018, home range size was similar for clinically positive individuals (mean ± SE; 45.1 ± 10.4 ha, n = 39) and for those without clinical signs (16.3 ± 4.8 ha, n = 15; F = 1.16, df = 1, 37, *p* = 0.29; Fig. 4). Daily distance travelled was also similar between clinically positive individuals (52 ± 5 m/day, n = 528 steps by 30 snakes) and those without clinical signs (52 ± 9 m/day, n = 187 steps by 12 snakes; *χ*^2^ = 0.19, df = 1, *p* = 0.66; Fig. 5).

For *P. vulpinus* swabbed in 2017 and 2018, home range size was similar for snakes that tested positive for *O. ophidiicola* (25.5 ± 5.5 ha, n = 13) and those on which the pathogen was not detected (13.4 ± 6.3, n = 8; F = 1.55, df = 1, 19, *p* = 0.23; Fig. 4). Individuals that tested positive for *O. ophidiicola* travelled longer daily distances (62 ± 10 m/day, n = 127 steps by 14 snakes) than snakes on which *O. ophidiicola* was not detected (33 ± 7 m/day, n = 89 steps by 8 snakes; *χ*^2^ = 10.25, df = 1, *p* = 0.001; Fig. 5).

## Discussion

We found no evidence for direct effects of ophidiomycosis on fitness in free-ranging *P. vulpinus,* despite this species exhibiting the highest prevalence of ophidiomycosis at our study site^34^. We did not observe a population decline over the study period, and clinically positive snakes did not have a lower survival probability, supporting the hypothesis that ophidiomycosis does not exert population-level effects. We recognize that we were likely studying a single strain of the pathogen at this site, and that ophidiomycosis may exert effects on fitness of species that do not occur at our site, or at sites with different environmental conditions^24,53^, although we are not aware of any robust examples of population-level effects in the current literature^19,33^. However, we did find evidence that individual variation in behaviour may affect risk of exposure to the pathogen.

Our mark-recapture analyses suggested fluctuations in population size among years, which is typical for many wildlife populations^3,54^, but did not indicate meaningful increases or declines over the study period (Table 1). This result is encouraging as this population is threatened by road mortality and habitat loss. Continued monitoring of this population can clarify whether it fluctuates on a particular cycle, and how environmental factors may drive these trends^3^.

Although clinically positive snakes did not experience reduced probability of survival, they may experience a higher rate of depredation (Fig. 2). Behavioural responses to infection may include increased risky behaviours such as basking that could increase exposure to predators such as raptors or raccoons (*Procyon lotor)*^55^, and *P. vulpinus* often basks in trees or shrubs^56–58^. The prevalence of ophidiomycosis in road-killed individuals was not higher than that observed in live snakes. However, species that bask terrestrially, rather than arboreally (e.g., *Sistrurus* spp.), often bask on roadsides to take advantage of increased heat absorbed by gravel and asphalt. Increased basking in response to infection may increase risk of road mortality in these species^59,60^. Further research is required to understand these potential indirect effects of ophidiomycosis on survival probability.

Animal movement can be influenced by a variety of factors, including environmental predictors^61^ and disease^16^. Ophidiomycosis was not associated with longer or shorter movements in our study, suggesting that infected snakes continued their typical behaviours (e.g., foraging, searching for mates). However, our results suggest that individual variation in movement behaviour might affect snakes’ risk of exposure to the pathogen. Snakes swabbed in 2017–2018, and on which *O. ophidiicola* was detected, had slightly larger home ranges than snakes not carrying detectable fungal loads. It is unlikely that these snakes moved farther because of exposure to the pathogen itself, particularly as clinically positive snakes did not. Instead, this result likely reflects how the risk of exposure to pathogens is affected by behavioural variation among individuals^62^. Snakes that move farther, and therefore contact a greater variety of substrates, are more likely to be exposed to *O. ophidiicola.* Larger home ranges are also more likely to overlap wetter habitats (e.g., marshes), in which snakes are more likely to carry detectable amounts of the fungus^23,34^.

Successful reproduction is an important component of fitness^63^, and ophidiomycosis did not appear to limit oviposition in our study. Pygmy Rattlesnakes (*Sistrusrus miliarus*) can produce viable offspring while affected with ophidiomycosis^64^, and we are unaware of any examples of the infection limiting reproduction success in other species. However, we used a coarse measure of reproductive success in this study as we were unable to monitor nest fate, hatch success, or the condition and survival rates of hatchlings. The potential long-term or carry-over effects of parental ophidiomycosis on offspring fitness deserve further consideration but would require intensive nest locating and monitoring.

Individual cases of severe ophidiomycosis are often associated with emaciation and loss of fat stores, which may contribute to eventual mortality^29,65^. Emaciation in these cases could result from anorexia, from oral lesions restricting feeding ability, or from lethargy restricting foraging effort^65^. However, McKenzie et al.^33^ found that body condition of *R. septemvittata* was not reduced in snakes with apparent ophidiomycosis, consistent with our observations of *P. vulpinus* in this study. Ophidiomycosis prevalence in the study population is highest at emergence from overwintering^34^, yet body condition of affected and unaffected groups was similar at emergence, and directly before overwintering.

However, subtle differences in body condition of affected and unaffected snakes did indicate potential behavioural impacts of ophidiomycosis. Male *P. vulpinus* with clinical signs had higher body condition than unaffected snakes in summer, concurrent with this species’ mating period (~ Day 140–161)^66^. We speculate that male snakes with ophidiomycosis may expend less energy searching for mates than males without ophidiomycosis, if they spend more time basking and moulting to clear the fungus. Accurate observation of mating frequency in free-ranging snakes is not feasible, but nest monitoring and subsequent paternity testing of successful nests could be used to test potential indirect effect of ophidiomycosis on male reproductive success, and to rigorously compare reproductive success among females as mentioned above.

While our results suggest that ophidiomycosis is unlikely to exert population-level effects, further research should explore potential fitness impacts in other species, as well as other factors that could alter host–pathogen interactions and disease severity. Anthropogenic threats such as climate change, pollution and the introduction of novel pathogens may interact with the effects of opportunistic pathogens with unpredictable cumulative effects on wildlife health^67,68^. Ophidiomycosis prevalence and severity may be exacerbated by wetter, cooler conditions^23,69–71^, and severe weather events are increasing in frequency with climate change^72^. Exposure to sub-lethal doses of some chemical contaminants can reduce immune function^68^ and co-infections (syndemics) can increase the severity of some fungal infections^73^. Climate change can also alter the distribution and impacts of pathogens and diseases^67^. The study of ophidiomycosis can benefit from a holistic approach that considers the prevalence and potential impact of the disease alongside other pressures on snake populations^74,75^.

## Data Availability

The datasets generated and analysed during the current study are available in the Open Science Framework repository, https://osf.io/jhsmb/.
